# Response of altitudinal vegetation belts of the Tianshan Mountains in northwestern China to climate change during 1989–2015

**DOI:** 10.1038/s41598-021-84399-z

**Published:** 2021-03-01

**Authors:** Yong Zhang, Lu-yu Liu, Yi Liu, Man Zhang, Cheng-bang An

**Affiliations:** Key Laboratory of Western China’s Environmental Systems (Ministry of Education), College of Earth and Environmental Sciences, Lanzhou University, Lanzhou, China

**Keywords:** Climate-change ecology, Climate change

## Abstract

Within the mountain altitudinal vegetation belts, the shift of forest tree lines and subalpine steppe belts to high altitudes constitutes an obvious response to global climate change. However, whether or not similar changes occur in steppe belts (low altitude) and nival belts in different areas within mountain systems remain undetermined. It is also unknown if these, responses to climate change are consistent. Here, using Landsat remote sensing images from 1989 to 2015, we obtained the spatial distribution of altitudinal vegetation belts in different periods of the Tianshan Mountains in Northwestern China. We suggest that the responses from different altitudinal vegetation belts to global climate change are different. The changes in the vegetation belts at low altitudes are spatially different. In high-altitude regions (higher than the forest belts), however, the trend of different altitudinal belts is consistent. Specifically, we focused on analyses of the impact of changes in temperature and precipitation on the nival belts, desert steppe belts, and montane steppe belts. The results demonstrated that the temperature in the study area exhibited an increasing trend, and is the main factor of altitudinal vegetation belts change in the Tianshan Mountains. In the context of a significant increase in temperature, the upper limit of the montane steppe in the eastern and central parts will shift to lower altitudes, which may limit the development of local animal husbandry. The montane steppe in the west, however, exhibits the opposite trend, which may augment the carrying capacity of pastures and promote the development of local animal husbandry. The lower limit of the nival belt will further increase in all studied areas, which may lead to an increase in surface runoff in the central and western regions.

## Introduction

Dynamic changes of altitudinal vegetation belts are often the consequence of climate change. As a special geomorphological unit, mountainous areas, due to their range of altitude and the influence of varied slopes and aspects, lead to diverse distributions of certain environmental factors in the vertical direction, which feature different characteristics and life forms^1^. For example, in the northwestern mountainous region of China, where the baseband is desert, as the altitude increases, the vegetation types gradually transition to montane steppe, coniferous forest and alpine meadow. Moreover, the temperature continues to decrease as the altitude increases, and precipitation increases at lower altitude and then decreasing at higher alttiudes^2^. The soil is also of distinct types and structures at different altitudes, slopes, and aspects. These factors change regularly in the vertical direction to form altitudinal vegetation belts of soil, climate, and vegetation^3^. In general, vegetation affects the organic matter content of the soil and the process of soil formation, and its response to climate change is relatively strong. Overall, its coverage and richness constitute important indicators for evaluating the ecological environment of mountainous areas.

The impact of climate change on alpine ecosystems has become a topic of focus in global change research^4,5^. Climate warming has led to an increase in global average surface temperature, sea-level rise, accelerated melting of glaciers in polar and high mountain areas, and frequent occurrence of extreme climate events^6,7^. Pertinent research has shown that the alpine ecosystem is relatively more sensitive to climate change, and alpine vegetation belts are highly dependent on temperature^8^. Therefore, temperature change exerts a significant effect on the dynamics of alpine vegetation belts. In addition, altitude is the main factor affecting the biodiversity and distribution of alpine ecosystems, and species richness decreases with increasing altitude^9^. Under the background of global warming, however, a tendency exists for low-altitude species to migrate to high altitudes in altitudinal belts of mountains^10–12^. These species primarily come from subalpine steppe or forest belts, resulting in an increase in the upper limit of the distribution of steppe belts and tree lines^13,14^. This trend of species migration has led to the emergence of low-altitude species adapted to the warm environment in alpine vegetation belts and the increase of species richness in high altitude areas^15^. Moreover, due to the ability of alpine plants or cushion-like vegetation to improve the living environment, the upward migration of species in low mountain belts has increased further^16^.

In the context of warming and humidification in northwest China^17^, determining how the Tianshan Mountain ecosystem responds could constitute a worthy topic. Extant literature has proven that the arid and semi-arid climate in northwestern China exhibits a clear trend of both warming and humidification^18^. The rise in surface temperature increases the instability of air convection, and produces increased evaporation and atmospheric water vapor, which may bring more precipitation to some areas^19^. As a huge east–west mountain series in the arid region of Central Asia, the Tianshan Mountains respond strongly to climate change. Many scholars have performed in-depth research on water resource issues resultant from climate change in Tianshan Mountains or Xinjiang province, China^20–22^. However, precisely how the altitudinal vegetation belts, as the most important environmental basis of the region, responds to regional and global climate change remains undefined. There is also a paucity of research focusing on whether consistent patterns exist in the response of different vegetation belts, or how changes in altitudinal vegetation belts affect human production activities. The current article attempts to fill in these gaps in the existing literature. We first utilized meteorological data to show the spatial differentiation of temperature and precipitation changes in the Tianshan Mountains from 1989 to 2015. Subsequently, the dynamics of the altitudinal vegetation belts were analyzed using remote sensing images. Finally, we closely examined the response of altitudinal vegetation belts dynamics to climate change and its impact on human activities. Based on the research results, we mainly discussed the impact of the dynamics of the altitudinal vegetation belts on agriculture and animal husbandry.

## Methods

### Study area

**Figure 1:**
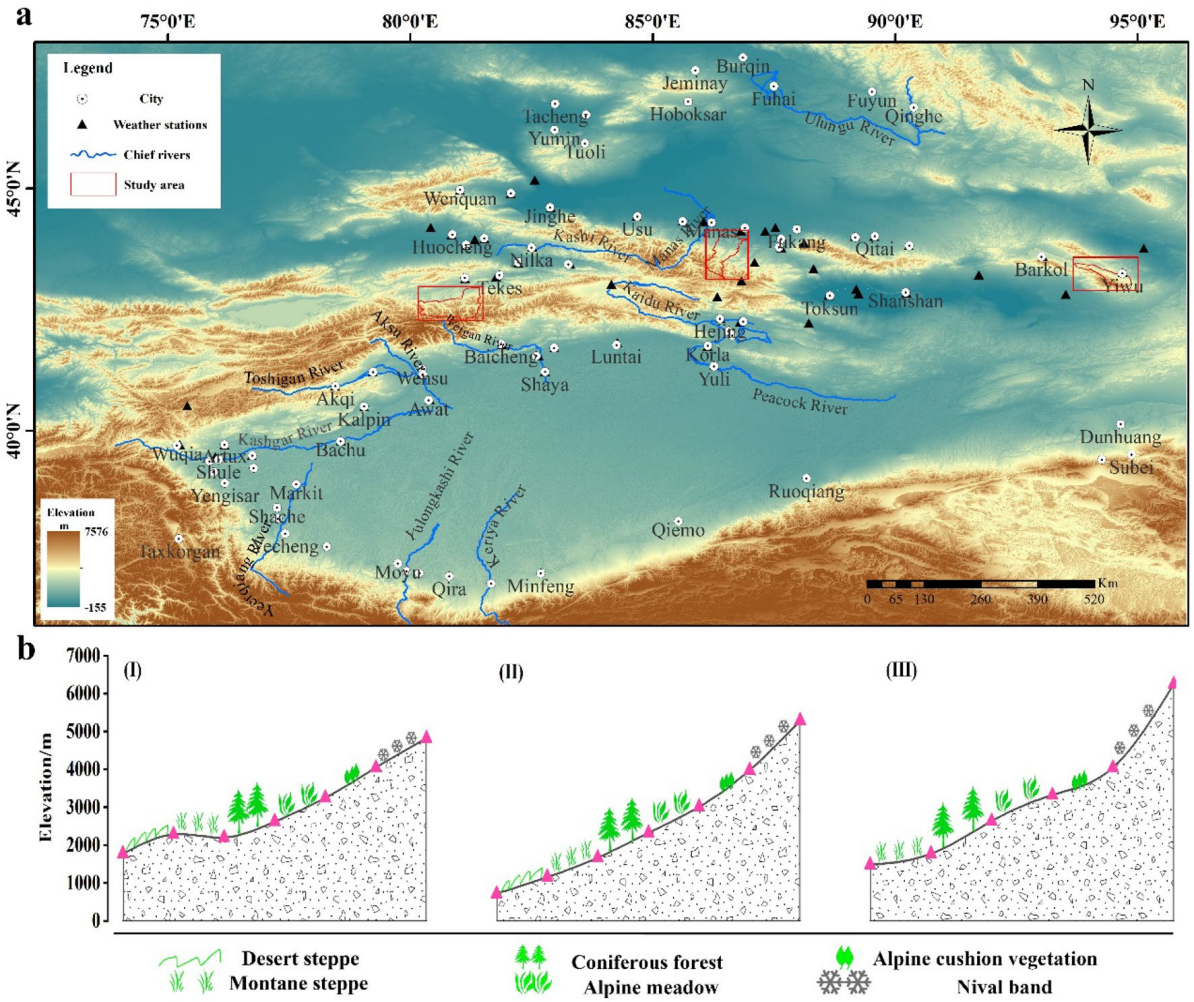
Location of the study area and the composition of the altitudinal vegetation belts. (**a**) The areas enclosed by the red rectangles are our study area. The rectangle on the left is Zhaosu county; the middle is Hutubi county; and the right is Yiwu county. (**b**) The altitudinal vegetation belts in Tianshan Mountains; (I), (II), and (III) indicate Yiwu county, Hutubi county, and Zhaosu county, respectively.

The Tianshan Mountains, northwest China, one of the seven major mountain systems in the world, is located in central Xinjiang, China. Tianshan Mountains is the largest mountain range in the world that is located in a temperate arid zone. It spans 1700 km from east to west, with an average elevation of approximately 2300 m. Its southern and northern parts comprise vast deserts. Because the area around Tianshan Mountains is located in the heartland of Eurasia, it is far from the ocean and forms a temperate continental climate. However, westerly winds from the Atlantic entered the Tianshan Mountains through the Caspian Sea and Central Asia, and brought abundant water vapor, which caused the Tianshan Mountains to have a unique humid climate, and thus is called the “Central Asia Wet Island”^23^. Precipitation in the Tianshan Mountains decreases from west to east. The average annual precipitation on the northern slope reaches 500 mm, and precipitation in the western forest zone can even reach 1100 mm. The most annual precipitation mainly occurs from May to June. Tianshan Mountains has rich and diverse natural landscapes, such as glaciers, permanent snow cover, virgin forests, grasslands and deserts, and thus forms the most complete mountain altitudinal vegetation belts in a temperate arid region in the world. In the eastern and central regions, as the altitude increases, it turns into a desert steppe belt, montane steppe belt, coniferous forest belt, alpine meadow belt, alpine cushion vegetation belt, and nival belt. In the western region, with the increase of altitude, it transitions into a montane steppe belt, coniferous forest belt, alpine meadow belt, alpine cushion vegetation belt, and nival belt (Fig. 1b) (part 1 in Supplementary Information). We selected Yiwu county in the east, Hutubi county in the middle and Zhaosu county in the west of the Tianshan Mountains as the study areas. We specifically aimed to extract the altitudinal vegetation belts of these areas, and elucidate the dynamics of altitudinal vegetation belts of the Tianshan Mountains (Fig. 1a).

### Dataset and data processing

In recent years, with the increasing frequency of satellite launches and rapid advancements in satellite imaging technology, the archiving of satellite data has greatly increased, which has promoted the application and development of satellite imaging in various fields^24–26^. Because remote sensing images have a high temporal resolution, spatial resolution and spectral resolution, they are widely utilized in monitoring vegetation and land cover changes. Therefore, we combined remote sensing images and decision tree classification methods to obtain the spatial distribution of the altitudinal vegetation belts of the Tianshan Mountains at different times (part 2 in Supplementary Information).

The remote sensing satellite data used in the study are Landsat 5/8 satellite data with a time period of 1989–2015. In the Tianshan Mountains, August to September constitutes the most prolific period of vegetation growth, and the annual biomass of different vegetation types reaches its maximum value during this time. As a consequence, we selected remote sensing images of these periods for the extraction of altitudinal vegetation belts. Between July and August, seasonal snow has completely melted, and only permanent snow and glaciers remain. Because permanent snow cover is relatively more sensitive to climate change, we chose remote sensing images from July to August to extract the nival belt. To obtain high quality remote sensing images, it is necessary to perform radiation correction, atmospheric correction, geometric correction, and clipping of the study area on these remote sensing images. Finally, the pre-processed remote sensing images were used to extract the altitudinal vegetation belts.

Digital elevation model (DEM) is used for the grid representation of terrain, and each pixel value represents the height from the datum. The extraction of altitudinal vegetation belts requires highly accurate DEM data. At present, international mainstream digital elevation models (DEMs) are mainly SRTM-DEM^27^, ASTER GDEM^28^, and AW3D30 DSM^29,30^. For this study, we chose to use AW3D30 DSM because it achieves both higher spatial resolution (30 m) and higher accuracy than other DEMs.

The meteorological data that we obtained included monthly average temperature and monthly precipitation, and the time range was from 1989 to 2015. These data were downloaded from the China Meteorological Administration (http://data.cma.cn/).

## Results

### Spatiotemporal differentiation of temperature and precipitation in the Tianshan Mountains

**Figure 2:**
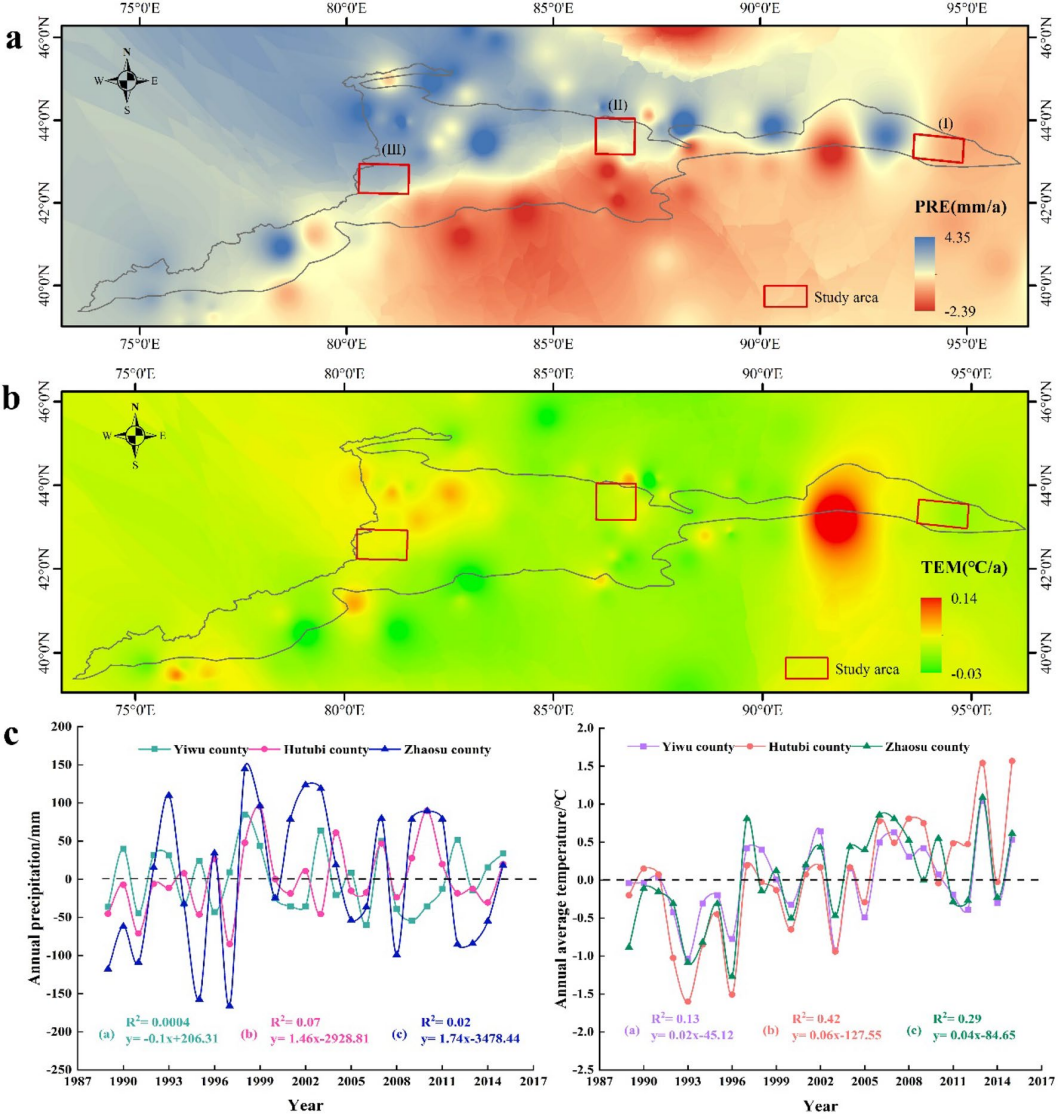
Spatial variation of temperature and precipitation [(**a**) indicated spatial variation of precipitation in Tianshan Mountains and surrounding areas; and (**b**) indicated spatial variation of temperature in Tianshan Mountains and surrounding areas]. (**c**) Indicated the changing trend of temperature and precipitation in the study area during 1989–2015. The Mann Kendall (MK) trend test was used to identify the climate change (i.e., temperature and precipitation) trend of the Tianshan Mountains during 1981–2015.

Figure 2 shows the spatial variation trend of annual precipitation and the annual average temperature in the Tianshan Mountains. The precipitation in the northern and western parts of the Tianshan Mountains showed an increasing trend, while precipitation in the southern and eastern parts showed a slight decreasing trend (Fig. 2a). Figure 2b presents the spatial variation trend of annual average temperature in the Tianshan Mountains. From the figure, one can discern that the temperature in most parts of the Tianshan Mountains is increasing, but the increase is spatially dissimilar. Especially in the eastern region, the increase was most significant. Moreover, the temperature in the eastern and western regions increased the most, while the central and southern regions exhibited the smallest increase in temperature. In general, the climate in the west and the north parts of the Tianshan Mountains showed a trend of “warm and humid”, but the climate in the south and the east showed a trend of “warm and dry”^31^ reported the following: the whole Tianshan Mountains present a warm and humid trend (1960–2016); the annual average temperature increase rate in the north slope is greater than that in the south slope; and annual precipitation has increased significantly in the north and west, but not in other areas. The spatial variation of temperature found in^31^ is in accordance with our research results. However, the changes in precipitation in the southern and eastern parts of the Tianshan Mountains differ from our results, which may be attributable to the different time frames of our respective investigations.

The temperature and precipitation trends in the study area are inconsistent (Fig. 2c). Precipitation in the eastern part (Yiwu county) exhibits a slightly decreasing trend, while precipitation in the central part (Hutubi county) and the western part (Zhaosu county) is increasing. Moreover, although the annual precipitation rates of increase in Hutubi county and Zhaosu county were 14.6 mm/10a (“10a” means per decade) and 17.4 mm/10a, respectively, they did not reach the significance level of 0.05. In addition, the average annual temperature in the study area is rising. Specifically, the average annual temperature increase rate of Hutubi county and Zhaosu county were 0.6 °C/10a and 0.4 °C/10a, respectively, and reached a significance level of 0.01. Although the temperature increase rate in Yiwu county was 0.2 °C/10a, it was found to be insignificant.

### Changes of altitudinal vegetation belts in the study area

**Figure 3:**
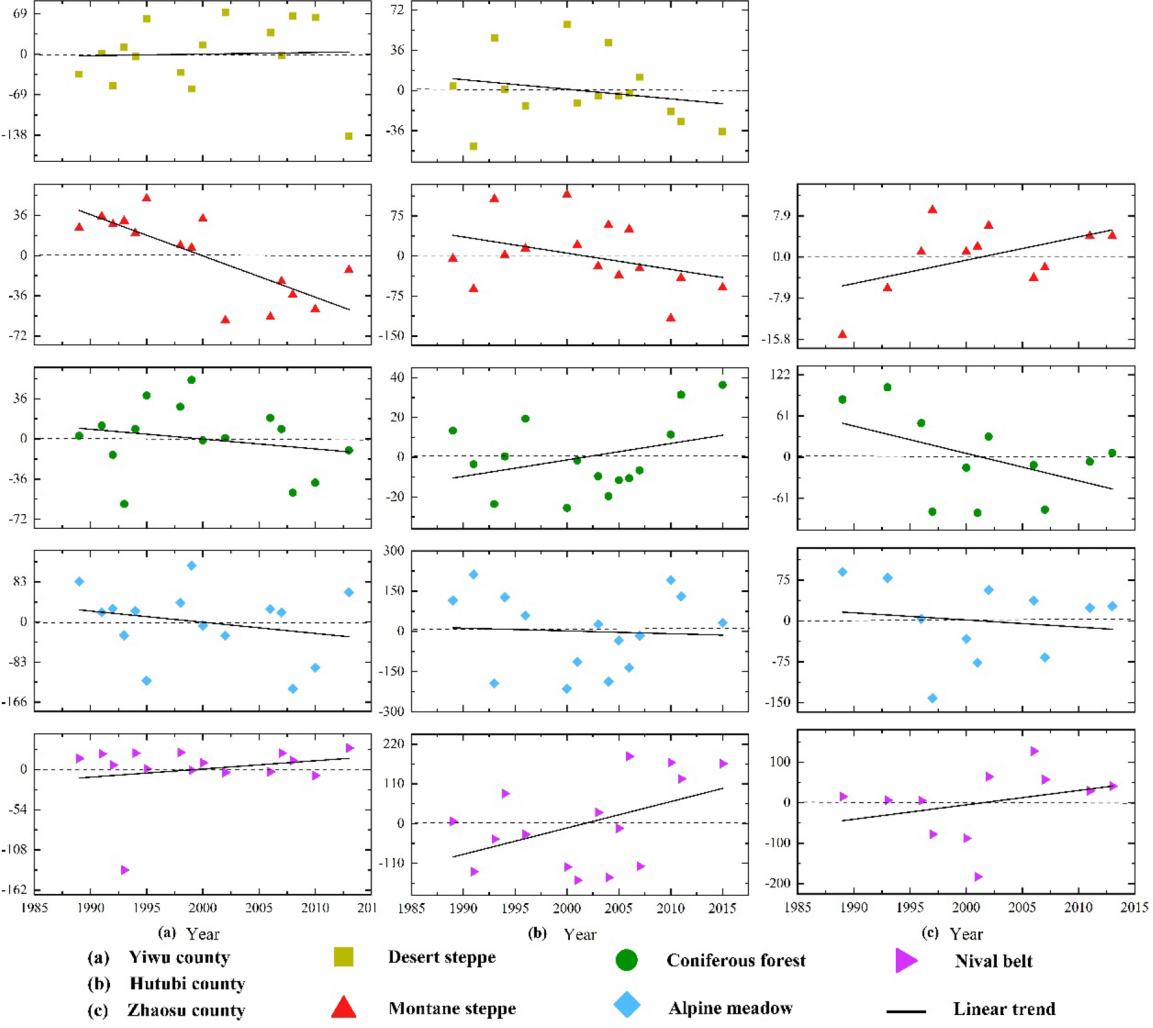
Changes of altitudinal vegetation belts in the study area from 1989–2015. All Y-axis represent anomalies values. Because the upper limit of the cushion vegetation is the lower limit of the nival belt, we only show the trend of the lower limit of the nival belt.

The upper limit of the montane steppe in Yiwu county (east) shift to lower altitudes was found to be the most significant. The altitudinal vegetation belts of Yiwu county also exhibited different trends (Fig. 3a). Although the upper limit of the desert steppe belt fluctuated greatly, the changing trend was non-obvious (i.e., a slight upward trend). The upper limits of the montane steppe, coniferous forests, and alpine meadows showed the same downward trend, and the downward trend of the montane steppe was determined to be comparably more significant. Furthermore, the lower limit of the nival belt exhibited a slight upward trend, and the upper limit of the cushion vegetation belt also showed an upward trend.

The lower limit of the nival belt in Hutubi county (central) had the most obvious upward trend. Figure 3b shows that the upper limits of the desert steppe, montane steppe, and alpine meadow show the same downward trend, and the downward trend of the montane steppe is more obvious. The nival belt, however, exhibits the opposite trend. By comparing the altitudinal vegetation belts at different altitudes, we find that the lower limit of the nival belt not only has a large inter-annual fluctuation, but also a more significant upward trend, which also demonstrates that the upper limit of cushion vegetation exhibits an obvious upward trend.

The upper limit of the montane steppe in Zhaosu county (western) had a clear upward trend. Warm and humid west winds from the Atlantic Ocean entered Yili Valley and brought abundant precipitation with the result that the montane steppe became the “baseband” of the altitudinal vegetation belts in the west of the Tianshan Mountains. Figure 3c reveals that the upper limit of the montane steppe features a significant upward trend, but the interannual fluctuation is small. In addition, the upper limits of coniferous forests and alpine meadows exhibit the same downward trend, and the trend of the coniferous forest belt is more obvious. Although the lower limit of the nival belt shows a small increase, the interannual fluctuation is obvious, which also demonstrates that the upper limit of the cushion vegetation belt exhibits an upward trend.

Through the above analysis, we found that the desert steppe belt has an opposite trend (expansion towards opposite altitudes) in the two sub-regions, and the trend is not significant. The montane steppe shows the same change trend in the east and middle, while the opposite change trend occurs in the west. Moreover, the changing trend of the montane steppe in all of the study areas is significant. Coniferous forests exhibit the same changing trend in the east and west, and the opposite trend in the middle. The upper limit of the alpine meadow belt features the same downward trend, but the changing trend is not obvious in all study areas. Furthermore, the lower limit of the nival belt shows the same upward trend in all study areas, and the changing trend in Hutubi County is more significant, which also demonstrates that the cushion vegetation belt exhibits the same change trend. Overall, the changing trend of the altitudinal vegetation belts in the low-altitude area of the Tianshan Mountains is different, but the changing trend of the altitudinal vegetation belts in the high-altitude area (above 3000 m) is the same.

### Response of different altitudinal vegetation belts to temperature and precipitation changes

In the monitoring time range, by comparing the altitudinal vegetation belts and the changing trend of precipitation, we found that only the change trend of the upper limit of the desert steppe belt in Yiwu county had an obvious relationship with annual precipitation change. In addition, precipitation in the three study areas did not exhibit a significant increasing or decreasing trend (Fig. 2c). These findings demonstrated that precipitation is not the main factor of altitudinal vegetation belts change.

**Figure 4:**
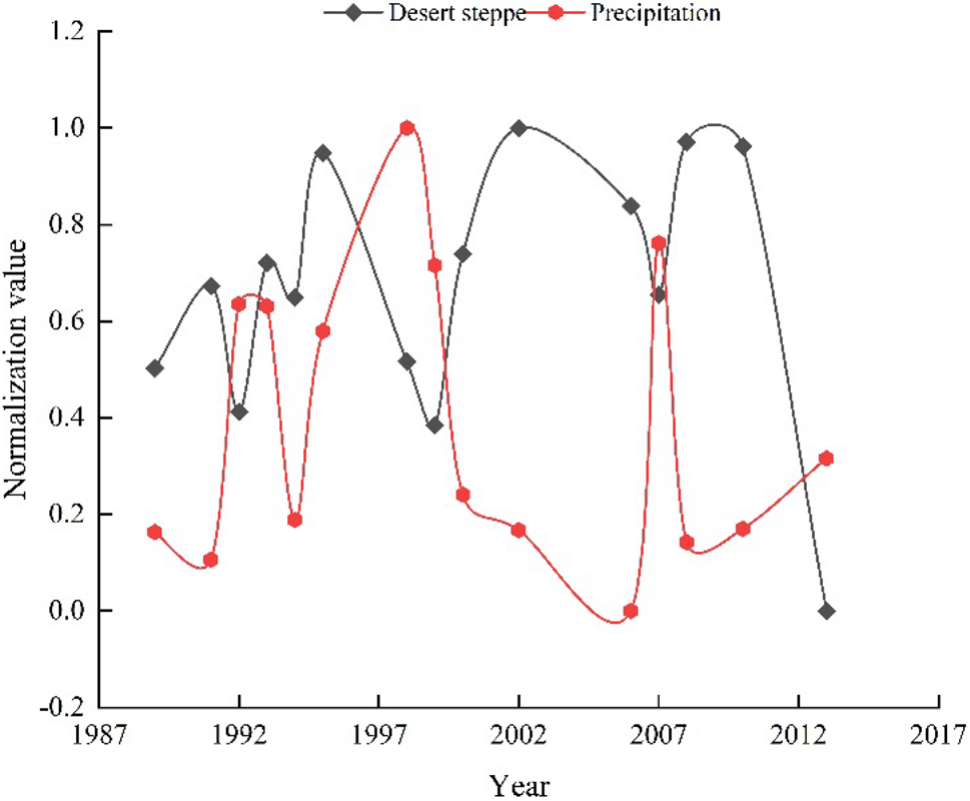
Temporal variation of precipitation. To better compare the variation trend of altitudinal vegetation belt, temperature and precipitation, we normalized the data of temperature, precipitation, and altitudinal belt spectrum. The normalized range of all data is [− 1, 1].

Figure 4 illustrates that the change of desert steppe and annual precipitation in Yiwu county has an opposite trend. The results indicate that, with the decrease of precipitation, the distribution of desert steppe may further expand. However, since there is no obvious decrease in precipitation, the distribution range of desert steppe will not expand significantly. This assertion is also supported by the fact that there is no obvious upward trend in the upper limit of the desert steppe (Fig. 3a).

**Figure 5:**
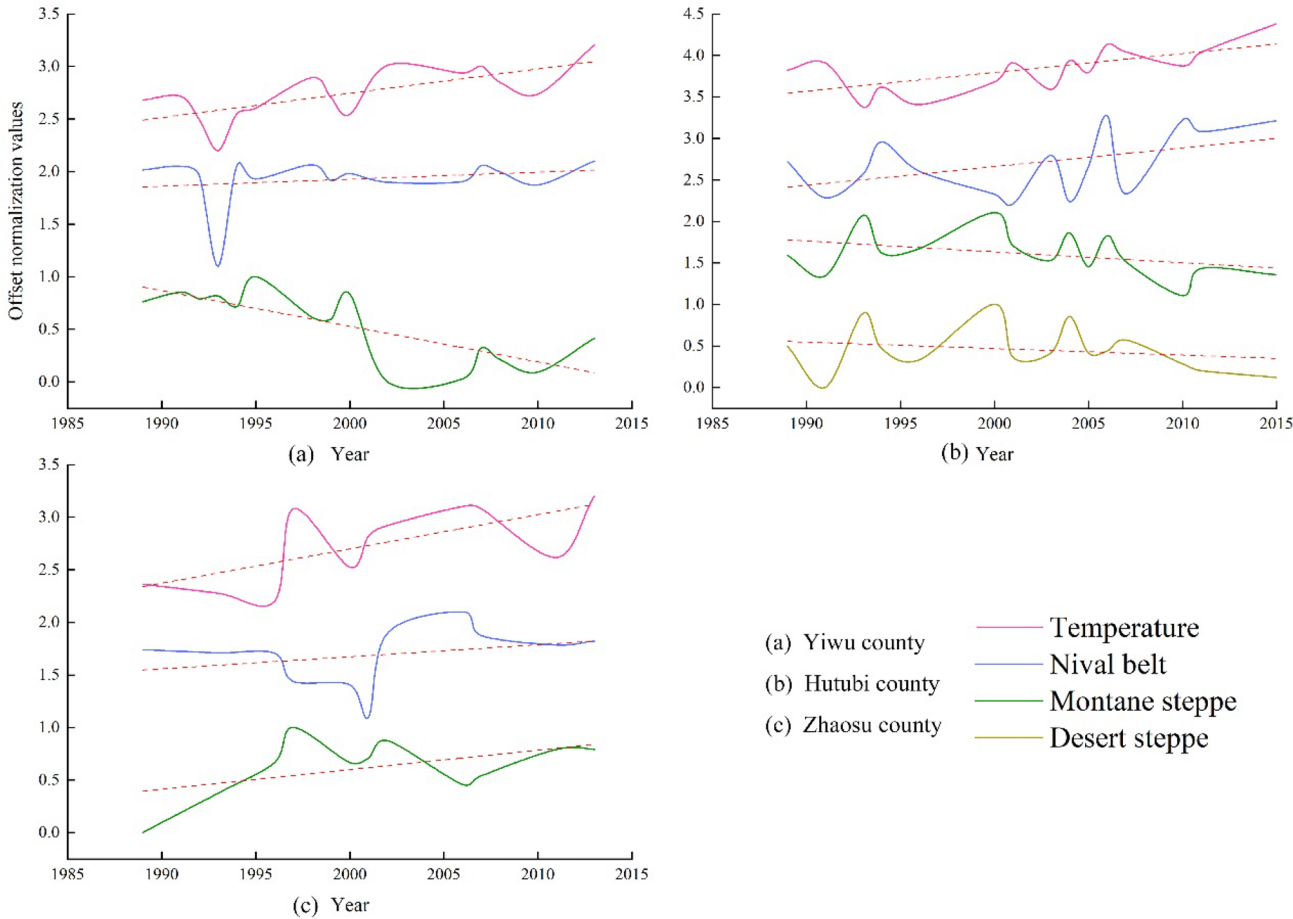
Temporal variation of temperature. [(**a**) Yiwu county; (**b**) Hutu bi county; and (**c**) Zhaosu county].

The steppe belts in different study areas exhibit different responses to temperature changes. Figure 5 shows that the changing trend of the montane steppe in the east (Yiwu county), and the desert steppe and montane steppe in the middle (Hutubi county), is opposite to the changing trend of annual average temperature. However, the changing trend of montane steppe and temperature in the west (Zhaosu county) is consistent. The results show that, with the increase of temperature, the upper limit of montane steppe in Yiwu county will be further reduced. Moreover, the desert steppe and montane steppe in Hutubi county also have the same trend. The upper limit of the montane steppe in Zhaosu county, however, will be further increased. Under the background of generally rising temperatures, the distribution range of montane steppe in Yiwu county, and desert steppe and montane steppe in Hutubi county, will decrease further, but the distribution range of montane steppe in Zhaosu county will continue to expand. In general, the montane steppe belt exhibits a clear response to changes in temperature, and temperature is the main factor of the change in the steppe belt.

The lower limit of the nival belt shows obvious responses to temperature changes in different study areas. Figure 5 illustrates that the lower limit of the nival belt and the temperature change trend are consistent in the three study areas. As the temperature increases, the lower limit of the nival belt will further increase, and the distribution range will narrow. These results indicate that temperature is the primary factor for the change of the nival belt. Furthermore, because the temperature rise rate is the largest in Hutubi county (Fig. 2c), the rising trend of the lower limit of the nival belt is more significant, which causes the range of the nival belt to more rapidly decrease. However, we found that the rate of temperature increase in Yiwu county was insignificant, which led to the shrinking of the nival belt being non-obvious. The changing trend of the nival belts in different study areas (Fig. 3) supports these conclusions.

The above analysis demonstrates that the altitudinal vegetation belts of the three study areas all exhibit a strong response to changes in temperature. Indeed, this study found that temperature constituted the main factor of altitudinal vegetation belts dynamics. The results also indicated that the montane steppe belt is more significantly affected by temperature than the nival belt. The general increase in temperature (Fig. 2c) will also lead to further shrinkage of snow coverage. However, due to the spatial difference in temperature in the nival belt, a different amplitude of increase was observed.

## Discussion

In the context of the coexistence of warm-wet and warm-dry climates in the Tianshan Mountains, we focused on which altitudinal vegetation belts dynamics exert an impact on human activities and how they respond to climate. Since snowmelt water is a critical water source for oases, low-altitude steppe belts are important locations for local herdsmen to develop animal husbandry. Consequently, we discussed the effects of these altitudinal vegetation belts on agriculture and animal husbandry.

### Influence of the change of altitudinal vegetation belts on agriculture and animal husbandry

The shrinking of the montane steppe in the eastern and central regions, as well as the expansion of the montane steppe in the western regions, have a potential impact on the development of local animal husbandry. In the montane steppe belt, high-cover vegetation needs to consume more water; whereas, in the desert steppe, sparse vegetation has a greater ability to resist drought. Therefore, the montane steppe is more sensitive to changes in moisture. The reason why the eastern mountain steppe belt shifts to low elevation is that less precipitation (104 mm/a) and rising temperatures promote more soil moisture to enter the atmosphere through evapotranspiration, which aggravates drought in low elevation regions. In the central region, the rate of temperature increase is the fastest (0.6 °C/10a), which enhances the instability of air convection, and results in a significant increase in local evapotranspiration^32^. These factors will lead to more severe droughts in low-elevation areas and exceed the tolerance of desert vegetation. Therefore, the montane steppe and desert steppe in the central region shrink toward low altitudes. The western region is located in the Yili Valley area with high precipitation (511 mm/a). In this region, the apparent increase in temperature leads to more water vapor entering the atmosphere, combined with the trumpet-shaped terrain and higher elevation (2027 m), which makes it easier to form precipitation. These conditions benefit the expansion of montane steppe.

The Tianshan Mountains have rich grassland resources due to its unique climatic conditions. Residents use this natural advantage to develop animal husbandry, and it has become one of their main agricultural production methods. In the Tianshan Mountains, a montane steppe is an important location for local herdsmen to graze. Changes in the montane steppe belt can greatly affect the scale of animal husbandry and the income of herdsmen. The degradation of the montane steppe in the east and central areas may impede the development of animal husbandry. The expansion of mountain grasslands in the west, however, will lead to improvement of the carrying capacity of grassland, which is conducive to better development of local animal husbandry.

In the Tianshan Mountains, glaciers-snow-melt water in high-altitude areas and precipitation in middle-altitude forest belts are the main sources of river supply, and the amount of river runoff has a major impact on agricultural production in oases^33^. Therefore, we suggest that the further narrowing of the distribution range of nival belts in different research areas of the Tianshan Mountains has a potential effect on downstream oasis agriculture. Indeed, the significant rising trend of temperature in the Tianshan Mountains has accelerated the rate of melting snow, causing the nival belts to shrink towards high altitudes. Furthermore, the reduction in snow cover reduces surface reflectance and leads to further reduction in snow cover. In particular, the significant increase of temperature in the middle of the Tianshan Mountains (Fig. 2c) causes the nival belt to shrink more rapidly towards higher elevation than other areas. The central and western regions also have higher snow cover^34^, combined with an increase in precipitation in the western region, which will benefit the development of oasis agriculture in the short-term. Although the melting rate of snow in the eastern region is high, the smaller glaciers, and snow cover area and precipitation (104 mm/a), will not cause a significant increase in runoff, and agricultural development in downstream oases may consequently be limited.

## Conclusions

Based on the meteorological data and remote sensing images, we analyzed the dynamic changes of vegetation altitudinal vegetation belts from three sub-regions of the Tianshan Mountains in Northwest China. The following main results were obtained.

The changes in the vegetation belts at low altitudes are spatially different. In high-altitude regions (higher than the forest belts), however, the trend of different altitudinal belts is consistent. The average annual temperature becomes the main factor for the change of the steppe belt and the nival belt.

The increase in temperature has caused the shrinkage of the steppe belt in Yiwu county and Hutubi county, which is not conducive to the development of animal husbandry in these areas. However, the expansion of the montane steppe in Zhaosu county will augment the livestock capacity of the pasture, which may produce favorable conditions for the development of local animal husbandry. The shrinkage of the nival belts in Hutubi county and Zhaosu county has also led to an increase in surface runoff, which is conducive to the development of downstream oasis agriculture in the short-term. However, there is less snow cover and precipitation in Yiwu county, resulting in water still constituting a limiting condition for agricultural development in downstream oases.

## Supplementary Information

Supplementary Information.

## References

[1] Sanchez-Gonzalez A, Lopez-Mata L (2005). Plant species richness and diversity along an altitudinal gradient in the Sierra Nevada, Mexico. Divers. Distrib..

[2] Dai L, Feng Y, Luo G, Li Y, Xu W (2014). The relationship between soil, climate and forest development in the mid-mountain zone of the Sangong River watershed in the northern Tianshan Mountains, China. J. Arid Land.

[3] Baiping Z, Ya T, Senguo MO (2004). Digital spectrum and analysis of altitudinal belts in the Tianshan Mountains. J. Mt. Res..

[4] Pretzsch H, Biber P, Schutze G, Uhl E, Rotzer T (2014). Forest stand growth dynamics in Central Europe have accelerated since 1870. Nat. Commun..

[5] Li WJ (2016). Effects of climate change on potential habitats of the cold temperate coniferous forest in Yunnan province, southwestern China. J. Mt. Sci. Engl..

[6] Donat MG, Lowry AL, Alexander LV, O'Gorman PA, Maher N (2017). Addendum: More extreme precipitation in the world's dry and wet regions. Nat. Clim. Change.

[7] Sun J, Qin XJ, Yang J (2016). The response of vegetation dynamics of the different alpine grassland types to temperature and precipitation on the Tibetan Plateau. Environ. Monit. Assess..

[8] Windmaisser T, Reisch C (2013). Long-term study of an alpine grassland: Local constancy in times of global change. Alpine Bot..

[9] Mahdavi P, Akhani H, Van der Maarel E (2013). Species diversity and life-form patterns in steppe vegetation along a 3000 m altitudinal gradient in the Alborz Mountains, Iran. Folia Geobot..

[10] Rumpf SB (2019). Extinction debts and colonization credits of non-forest plants in the European Alps. Nat. Commun..

[11] Lamprecht A, Semenchuk PR, Steinbauer K, Winkler M, Pauli H (2018). Climate change leads to accelerated transformation of high-elevation vegetation in the central Alps. New Phytol..

[12] Lenoir J, Gegout JC, Marquet PA, de Ruffray P, Brisse H (2008). A significant upward shift in plant species optimum elevation during the 20th century. Science.

[13] Kueppers LM (2017). Warming and provenance limit tree recruitment across and beyond the elevation range of subalpine forest. Glob. Change Biol..

[14] Sedmakova D (2019). Growth-climate responses indicate shifts in the competitive ability of European beech and Norway spruce under recent climate warming in East-Central Europe. Dendrochronologia.

[15] Fadrique B, Feeley KJ (2016). Commentary: Novel competitors shape species' responses to climate change. Front. Ecol. Evol..

[16] Cavieres LA (2014). Facilitative plant interactions and climate simultaneously drive alpine plant diversity. Ecol. Lett..

[17] Li BF, Chen YN, Chen ZS, Xiong HG, Lian LS (2016). Why does precipitation in northwest China show a significant increasing trend from 1960 to 2010?. Atmos. Res..

[18] Peng DD, Zhou TJ (2017). Why was the arid and semiarid northwest China getting wetter in the recent decades?. J. Geophys. Res. Atmos..

[19] Hong CP (2019). Impacts of climate change on future air quality and human health in China. Proc. Natl. Acad. Sci. U.S.A..

[20] Xu CC, Chen YN, Chen YP, Zhao RF, Ding H (2013). Responses of surface runoff to climate change and human activities in the arid region of Central Asia: A case study in the Tarim River Basin, China. Environ Manag..

[21] Deng HJ, Chen YN, Wang HJ, Zhang SH (2015). Climate change with elevation and its potential impact on water resources in the Tianshan Mountains, Central Asia. Glob. Planet. Change.

[22] Luo M (2019). Identifying climate change impacts on water resources in Xinjiang, China. Sci. Total Environ..

[23] Yue XY, Liu G, Chen JM, Zhou CY (2020). Synergistic regulation of the interdecadal variability in summer precipitation over the Tianshan mountains by sea surface temperature anomalies in the high-latitude Northwest Atlantic Ocean and the Mediterranean Sea. Atmos. Res..

[24] Zhang HKK, Roy DP (2017). Using the 500 m MODIS land cover product to derive a consistent continental scale 30 m Landsat land cover classification. Remote Sens. Environ..

[25] Hu ZY, Dietz A, Kuenzer C (2019). The potential of retrieving snow line dynamics from Landsat during the end of the ablation seasons between 1982 and 2017 in European mountains. Int. J. Appl. Earth Obs..

[26] Geng LY, Che T, Wang XF, Wang HB (2019). Detecting spatiotemporal changes in vegetation with the BFAST model in the Qilian Mountain region during 2000–2017. Remote Sens. Basel.

[27] Pham HT, Marshall L, Johnson F, Sharma A (2018). A method for combining SRTM DEM and ASTER GDEM2 to improve topography estimation in regions without reference data. Remote Sens. Environ..

[28] Piloyan A, Milan K (2017). Semi-automated classification of landform elements in Armenia based on SRTM DEM using K-means unsupervised classification. Quaest. Geogr..

[29] Gonzalez-Moradas MDR, Viveen W (2020). Evaluation of ASTER GDEM2, SRTMv3.0, ALOS AW3D30 and TanDEM-X DEMs for the Peruvian Andes against highly accurate GNSS ground control points and geomorphological-hydrological metrics. Remote Sens. Environ..

[30] Florinsky I, Skrypitsyna T, Luschikova O (2018). Comparative accuracy of the AW3D30DSM, ASTER GDEM, and SRTM1 DEM: A case study on the Zaoksky testing ground, Central European Russia. Remote Sens. Lett..

[31] Xu M, Kang SC, Wu H, Yuan X (2018). Detection of spatio-temporal variability of air temperature and precipitation based on long-term meteorological station observations over Tianshan Mountains, Central Asia. Atmos. Res..

[32] Wu P, Ding YH, Liu YJ, Li XC (2019). The characteristics of moisture recycling and its impact on regional precipitation against the background of climate warming over Northwest China. Int. J. Climatol..

[33] Lutz AF, Immerzeel WW, Shrestha AB, Bierkens MFP (2014). Consistent increase in High Asia's runoff due to increasing glacier melt and precipitation. Nat. Clim. Change..

[34] Chen YN, Li WH, Deng HJ, Fang GH, Li Z (2016). Changes in Central Asia's water tower: Past, present and future. Sci. Rep..

