# Supplementary material for: Response of altitudinal vegetation belts of the Tianshan Mountains in northwestern China to climate change during 1989–2015
Source: Sci Rep. 2021 Mar 1;11:4870. doi: 10.1038/s41598-021-84399-z (PMC7921417; doi:10.1038/s41598-021-84399-z)
Supplement: Supplementary file 1 — Supplementary Information. [file 41598_2021_84399_MOESM1_ESM.docx]

**Response of altitudinal vegetation belts of the Tianshan Mountains in northwestern China to climate change during 1989-2015**

Yong Zhang., Lu-yu Liu., Yi Liu., Man Zhang., Cheng-bang An*

**Supplementary Information**

**Part 1. Supplementary Photos**

- 1. **Desert steppe**

The desert steppe, as the baseband of the altitudinal vegetation bands, is located in the low altitude area of Tianshan Mountains in northwest China (Figure S1a). Here, the annual precipitation is less than 200mm, and mainly xerophyte plants such as *Stipa caucasica* and *Stipa tianschanica* grow. The width of the altitude they occupy is between 300-400m. The vegetation coverage is significantly less than the mountain steppe.

- 1. **Montane steppe**

Montane steppe is mainly distributed in the middle and low altitude areas (about 1500-2200m) on the northern slope of the Tianshan Mountains, and are distributed in more humid areas than desert grasslands (Figure S1b). It is located between a desert steppe and a coniferous forest. Here, dominant plants mainly include *Stipa capillata*, *Helictotrichon tianschanicum*, *Geranium wilfordii*, and so on.

- 1. **Coniferous forests**

Coniferous forests composed of *Picea schrenkiana* are mainly distributed on shady slopes and the elevation range is mainly between 1600-2800m (Figure S1c). The annual precipitation here is between 400-600mm. Besides, coniferous forests are suitable for growing in cold and humid climates.

- 1. **Alpine meadow**

Alpine meadows mainly include cold-resistant perennial herbs and various wormwoods are the main constructive plants (Figure S1d). It is mainly distributed between 2400-3900m above sea level. Here, the annual precipitation is between 400-500mm.

- 1. **Alpine cushion vegetation**

Alpine cushion vegetation is mainly distributed in alpine belts above 3000m above sea level. They adapt to the special ecological environment and are distributed in the shape of patches at high altitudes (Figure S1e). The plants here have an obvious xeromorphic structure, and the plants are short. Dense stems and branches form a cushion shape and spread on the ground. Here, it is dry in summer but rich in precipitation in winter. In general, the plants here can adapt to harsh climatic environments and are more sensitive to climate change.

- 1. **Nival band**

Nival band is composed of permanent snow and glaciers, and the height of the snow line is mainly between 3600-4200m (Figure S1f).

**
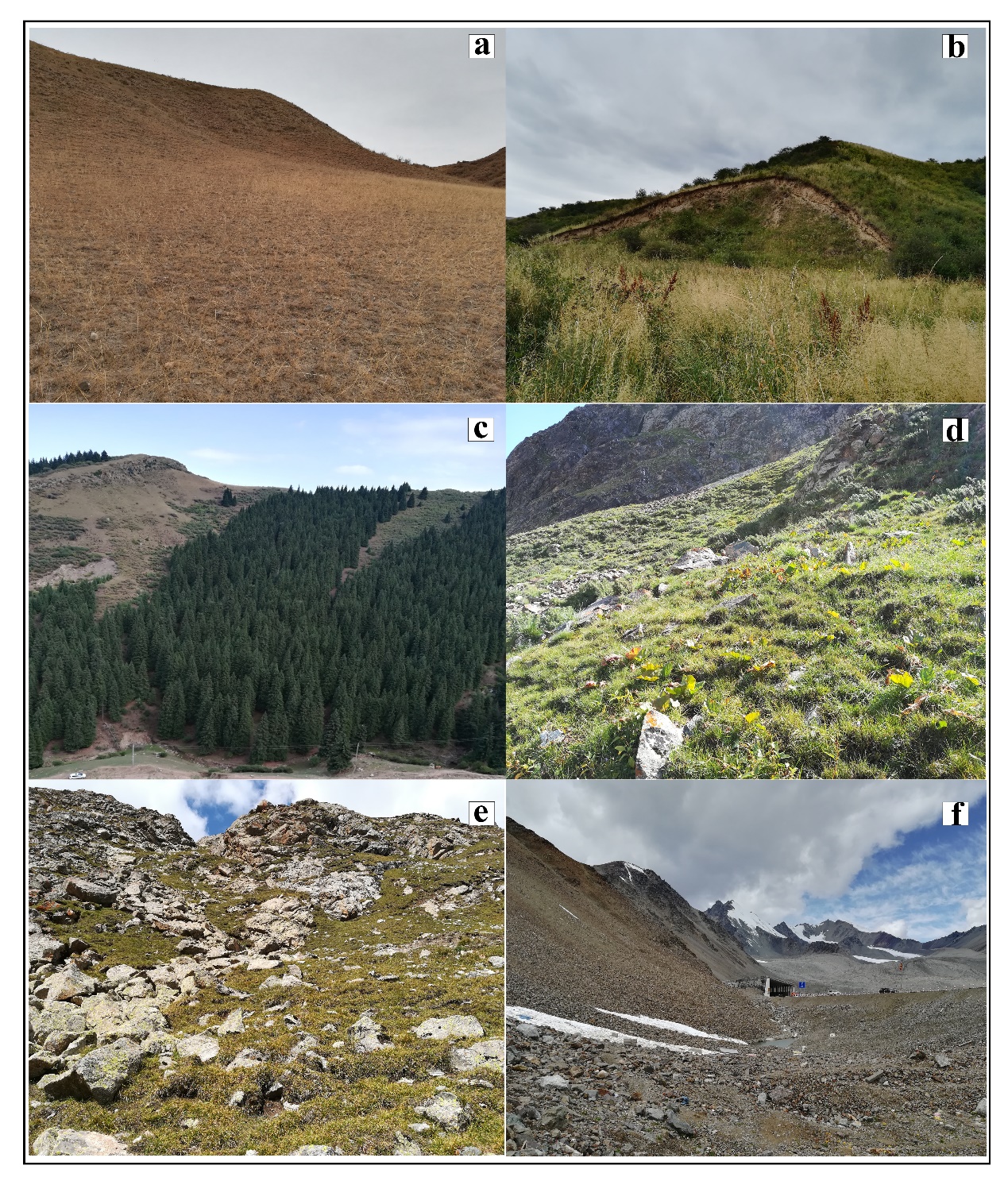
**

**Figure S1.** Altitudinal vegetation bands of Tianshan Mountains in Northwestern China. a: Desert steppe. b: Montane steppe. c: Coniferous forests. d: Alpine meadow. e: Alpine cushion vegetation. f: Nival band.

**Part 2. Supplementary methods**

**2.1. Vegetation indices calculation**

To ensure extraction accuracy of the altitudinal belt spectrum, we chose NDVI (normalized difference vegetation index)^1,2^ and NDSI (normalized difference snow index)^3^ to apply the decision tree classification process. The formula is as follows:

$NDVI=\frac{b_{nir}-b_{red}}{b_{nir}+b_{red}}$ (1)

$NDSI=\frac{b_{green}-b_{swir1}}{b_{green}+b_{swir1}}$ (2)

where b_nir_ is the near-infrared band; b_red_ is the red band; b_green_ is the green band; and b_swir1_ is the short-wave infrared band. NDVI can reflect the growth status and vegetation coverage of mountain vegetation, and achieves high accuracy in dynamic vegetation monitoring^4^. NDSI combines the short-wave infrared band and green band to highlight snow information in the image, which can effectively identify snow coverage^5^.

**2.2. Decision tree classification method**

The decision tree classification method offers the advantages of high data processing efficiency, short running time, and high accuracy of classification results^6,7^. For these reasons, this study employed the decision tree classification method to extract the altitudinal vegetation bands of the Tianshan Mountains. Afterwards, we verified the reliability of this method. The detailed classification process is presented in the following.

Snow cover is mainly extracted through NDSI. Areas where NDSI> 0.4 are generally considered to be snow. The NDSI values of snow and water bodies, however, can have overlapping parts. We compared their reflectivity values in the near-infrared band, and found that the near-infrared band can effectively differentiate snow from water bodies within a reasonable threshold. Therefore, we set NIR≥1000 as snow cover and NIR <1000 as a water body (Figure. S2).

**
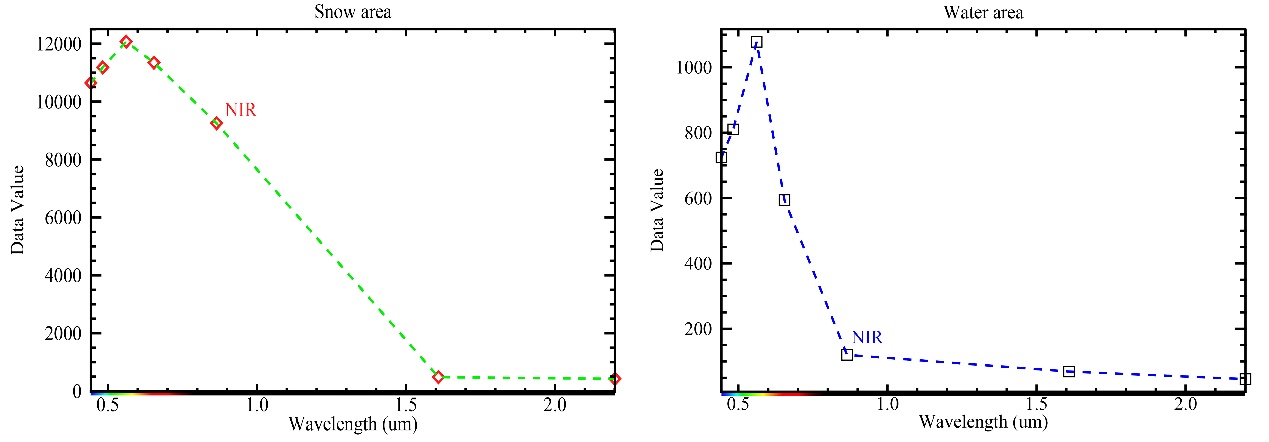
**

**Figure S2.** Spectral curves of snow and water. NIR is the near-infrared band of remote sensing images obtained from Landsat. (https://earthexplorer.usgs.gov/).

NDVI can distinguish desert grassland and cushion vegetation from other vegetation types, and DEM data can effectively differentiate desert steppe and cushion vegetation. Combining Google Earth™ and field investigation data, we regard areas with NDVI <0.3 and DEM <1800 as desert grassland, and areas with NDVI <0.3 and DEM> 2500 as cushion vegetation.

The NDVI ranges of coniferous forests and montane steppes are similar. By selecting representative samples of these two vegetation types, however, we determined that the coniferous forest exhibits strong absorption characteristics in the short-wave infrared band, while the mountain grassland possesses strong reflection characteristics (Figure S3). Finally, we combined NDVI (coniferous forest NDVI is generally greater than 0.5) and short-wave infrared spectral features to extract montane steppes and coniferous forests.


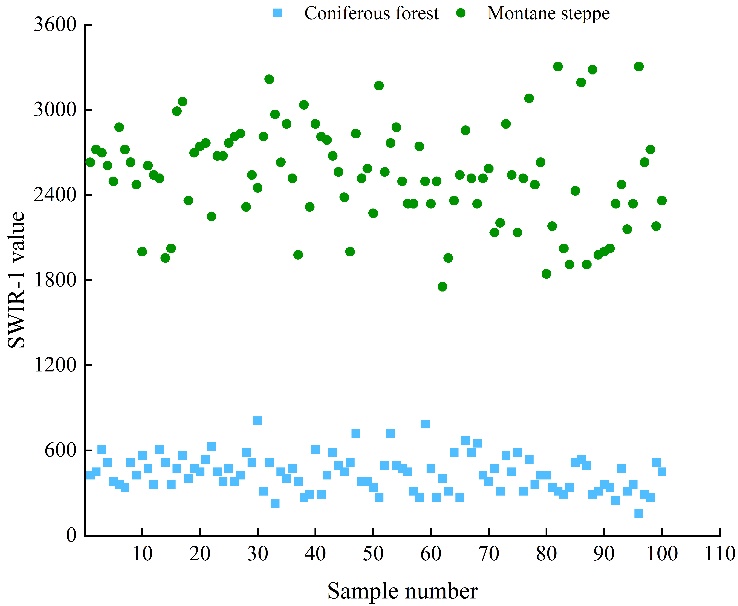


**Figure S3.** Reflectance values of coniferous forests and montane steppe.

Because alpine meadows and montane steppes possess similar spectral characteristics, NDVI cannot effectively distinguish between these two types of vegetation. However, the distribution of the two vegetation types differs in elevation. By analyzing field survey data, we conclude that the NDVI of both vegetation types is greater than 0.3. Moreover, if the altitude is above 2000 m, it is an alpine meadow; otherwise, it is a montane steppe.

**2.3. Precision of extraction results**

We selected the 2015 classification results as the verification object. The field survey data and Google Earth^™^ were utilized to obtain the sample points in different altitudinal vegetation bands to evaluate the reliability of this method in extracting altitudinal vegetation bands. The accuracy verification results demonstrate that the overall classification accuracy is 76.55%, and the Kappa coefficient is 0.7. The achieved classification accuracy is shown to be satisfactory.

**References**

1. Wang, X., Jin, R., Du, J. P. & Liang, H. Trend of surface freeze-thaw cycles and

vegetation green-up date and their response to climate change on the Qinghai-Tibet Plateau. *Journal of Remote Sensing.* **22**, 508-520 (2018). (in Chinese)

1. Tang, B. H., Shao, K., Li, Z. L., Wu, H. & Tang, R. An improved NDVI-based threshold method for estimating land surface emissivity using MODIS satellite data. *Int J Remote Sens.* **36**, 4864-4878 (2015).
2. Bulley, H. N. N., Bishop, M. P., Shroder, J. F. & Haritashya, U. K. Integration of

classification tree analyses and spatial metrics to assess changes in supraglacial lakes in the Karakoram Himalaya. *Int J Remote Sens.* **34,** 387-411 (2013).

1. Cai, Z. Z., Jonsson, P., Jin, H. X. & Eklundh, L. Performance of smoothing methods

for reconstructing NDVI time-series and estimating vegetation phenology from MODIS data. *Remote Sens-Basel.* **9**, 1271 (2017).

1. Mityok, Z. K., Bolton, D. K., Coops, N. C., Berman, E. E. & Senger, S. Snow cover

mapped daily at 30 meters resolution using a fusion of multi-temporal MODIS NDSI data and Landsat surface reflectance. *Can J Remote Sens.* **44**, 413-434 (2018).

1. Sun, Z. C., Leinenkugel, P., Guo, H. D., Huang, C. & Kuenzer, C. Extracting

distribution and expansion of rubber plantations from Landsat imagery using the C5.0 decision tree method. *J Appl Remote Sens.* **11**, 026011 (2017).

1. Dietz, A. J., Kuenzer, C., Gessner, U. & Dech, S. Remote sensing of snow - a review of available methods. *Int J Remote Sens.* **33**, 4094-4134 (2012).
